# From concept to practice: Patient safety culture and patient safety climate in dentistry

**DOI:** 10.1038/s41405-026-00500-6

**Published:** 2026-11-04

**Authors:** Eduardo Ensaldo-Carrasco, Jacqueline Adelina Rodríguez-Chávez, María Luisa Peralta-Pedrero, Nermin Tarhan, Carlos Aceves-González

**Affiliations:** 1 Centro de Investigaciones en Ergonomía, Centro Universitario de Arte, Arquitectura y Diseño (CUAAD), Universidad de Guadalajara (UDG), Guadalajara, Jalisco México; 2 School of Dentistry, Centro Universitario de Ciencias de la Salud, Universidad de Guadalajara (UDG), Guadalajara, Jalisco Mexico; 3 School of Medicine, Universidad Nacional Autónoma de México (UNAM), Av. Universidad 3000, Mexico City, Mexico; 4 Department of Periodontology, Faculty of Dentistry, Hacettepe University, Sıhhiye, Ankara Turkey; 5 School of Design & Creative Arts, Loughborough University, Loughborough, UK

**Keywords:** Dental public health, Special care dentistry

## Abstract

Research on safety culture and safety climate in dentistry is limited, leading to gaps in understanding and implementation. Therefore, this literature review examined the significance of patient safety culture and safety climate in dentistry, emphasizing their contributions to enhancing the quality of dental care and mitigating adverse events. Patient safety lapses in dentistry include mistakes in procedures, diagnoses, communication, and equipment, and they are often caused by people, systems, and the environment. Safety culture refers to the rules and guidelines that govern how a healthcare organization is structured, while safety climate refers to the attitudes and behaviors common in everyday practice. These ideas are connected, so they need to be addressed together. Essential strategies to enhance safety culture and climate include (1) leadership; (2) education and training for personnel and patients; (3) effective communication and non-punitive reporting mechanisms; (4) standardization through protocols and checklists; (5) teamwork and collaboration; (6) patient engagement; (7) technological advancements such as electronic health records and incident reporting systems; and (8) continuous improvement through audits and performance metrics. To make dentistry safer for patients, we need to move from separate treatments to more unified ones. The field of dentistry can move beyond reactive measures and create a culture where safety is a top priority for everyone by making it part of organizational principles, everyday practices, and patient collaboration.

## Introduction

Healthcare providers play a vital role in guaranteeing that all healthcare services are rendered with the highest quality and safety standards [1]. However, it is unfortunate that adverse events happen in healthcare facilities in about 10% of hospitalizations [2]. Two percent to 3% of consultations in primary care services are patient safety incidents, which are events that happen before bad things happen [3]. So, healthcare providers, including dentists, must always be on the lookout for ways to keep high standards of quality and safety, cut down on these kinds of incidents, and make sure their patients get the best care possible.

The World Health Assembly (WHA) has recognized adverse events as a public health problem since 2002, as reflected in Resolution WHA55.18 [4]. Since then, member states of the World Health Organization (WHO) have collected empirical evidence on incidents and adverse events and have developed and implemented strategies to reduce them. These include creating incident reporting systems [5], a conceptual framework to foster a standardized terminology [6], patient safety curricula [7], and encouraging a culture of safety [8]. However, the WHA has expressed concern, through Resolution WHA72.6, since these strategies have not shown a significant impact globally [9]. A reason for this is that most of the research activity on patient safety has been done in hospitals in developed economies [10], which shows that these strategies have not been adapted and executed in developing economies.

Over the last ten years, the growth of the field of patient safety in primary care has been slow. The systematic review by Lawati et al. [11] showed that, after reviewing the literature on patient safety for both hospital and primary care from 2000 to 2014, the focus on primary care began to emerge between 2011 and 2012 [11]. Yet, patients usually go to primary care facilities first before seeking hospital care. So, more patients are treated in these settings, and therefore mistakes are more likely to happen, and therefore, the greater the opportunity to analyze them. Although studies comparing these incidents across hospitals and primary care settings have not been conducted, it appears that diagnostic and judgment-related errors may be more common than reported in hospitals [12]. However, in contrast to hospitals and primary healthcare services, research on patient safety in dentistry is more limited [13] and therefore, strategies are needed to improve it (Table 1). As Pemberton et al. [14] suggested [14], one of these is to develop and maintain a patient safety climate within the work environment of dental practices. However, this concept is often misunderstood and may limit its implementation in dentistry, as it is sometimes confused with the concept of patient safety culture. Therefore, we aim to examine these concepts, their significance, and their role in improving the quality of oral health care and reducing adverse events.

**Table 1:** Strategies for Improving Patient Safety in Dentistry.

1. Identification of patient safety threats through incident reporting
2. Evaluation of incidents and identification of best practices
3. Communication and education about patient safety
4. Development of a patient safety climate

## Patient safety culture and patient safety climate

Halligan et al. [15], Weaver et al. [16], Churruca et al. [17], and Vikan et al. [18] conducted systematic reviews reporting that these terms are often used interchangeably [15–18], in which “patient safety culture” is the most frequent term. However, previous studies have examined their differences and established their corresponding methodological frameworks [15, 19–21]. For instance, the rules, beliefs, and practices within a healthcare system or organization related to patient safety constitute a culture of patient safety [15, 17]. People often use qualitative methods, such as in-depth interviews or focus groups, to measure this idea because it is complex and usually reflects a high-level management point of view [15, 20]. In contrast, patient safety climate refers to the temporary, subjective aspect of patient safety that depends on the current situation, context, and expectations of a healthcare team or unit [15, 22]. It represents a shared concept that emerges from individual perspectives on how important safety is within the workplace [23, 24]. It is commonly assessed from a low-level management perspective and usually uses quantitative methods to measure healthcare professionals’ views on procedures, practices, and behaviors [15, 22]. Questionnaires are frequently used to measure this concept, reflecting the patient safety culture at a specific point in time [24, 25]. Therefore, there are clear distinctions between these concepts, yet there is no broad consensus on the best way to measure safety culture and safety climate, even though they are not the same. The most common term is “patient safety culture,” but it is measured in the same way as “patient safety climate.”

## Importance of patient safety culture

Oral healthcare involves conducting many different procedures, materials, equipment, and technologies that interact closely with patients. These factors pose numerous potential risks, putting both patients and dental personnel at risk due to errors in their interactions. From a patient safety standpoint, incidents and adverse events in dentistry may cause physical, psychological, and financial damage to patients. These events can also harm the reputation and integrity of dental practitioners and institutions [26].

Patient safety incidents (PSIs) and adverse events can occur at any stage of providing oral healthcare. These events can happen at any point during the process of providing oral healthcare. The literature broadly classifies PSIs into delays in treatment, procedural errors, medication errors, wrong-tooth extractions, postoperative complications, diagnostic errors, communication failures, and equipment failures [13, 27–30]. However, research on patient safety is not as well developed as the progress made in medicine [13, 31, 32]. There are several reasons why it has taken longer for patient safety to be recognized. These include a historical focus on technical skill over systemic safety, the perception of lower risk, the absence of incident-reporting systems, restricted data collection, and professional autonomy in dental care, particularly in private practices where these services are predominantly provided [32]. Nevertheless, the empirical evidence in dentistry, comparable to that from other primary care services, suggests that potential contributing factors to these safety lapses include human error, systemic deficiencies, environmental influences, equipment failure, and an inadequate organizational culture [27, 29].

Human-factor failures include fatigue, insufficient training, cognitive overload, and communication errors [27]. These are closely linked to clinicians’ well-being, as dental practice imposes sustained cognitive demands. Time pressure alone has been shown to reduce dentists’ diagnostic accuracy [33]; in turn, high job strain and long working hours contribute to burnout among dental professionals [34, 35]. Burnout and emotional exhaustion have been associated with poorer patient safety across healthcare [36] and with dentists’ concern about having made errors [37], suggesting that declining well-being may impair clinicians’ cognitive and physical performance and put the safety and quality of dental procedures at risk. Systemic problems include inadequate systems for tracking patient records, scheduling, inefficient workflows, and a lack of follow-up [13, 38]. Poorly designed workspaces and insufficient resources are two examples of environmentally related factors [39]. Equipment-related flaws that can lead to safety lapses are caused by faulty equipment or poor maintenance [27]. Lastly, the weak organizational culture of dental care services may be attributed to poor teamwork and a lack of focus on patient safety within dental practices [38].

**Fig. 1:**
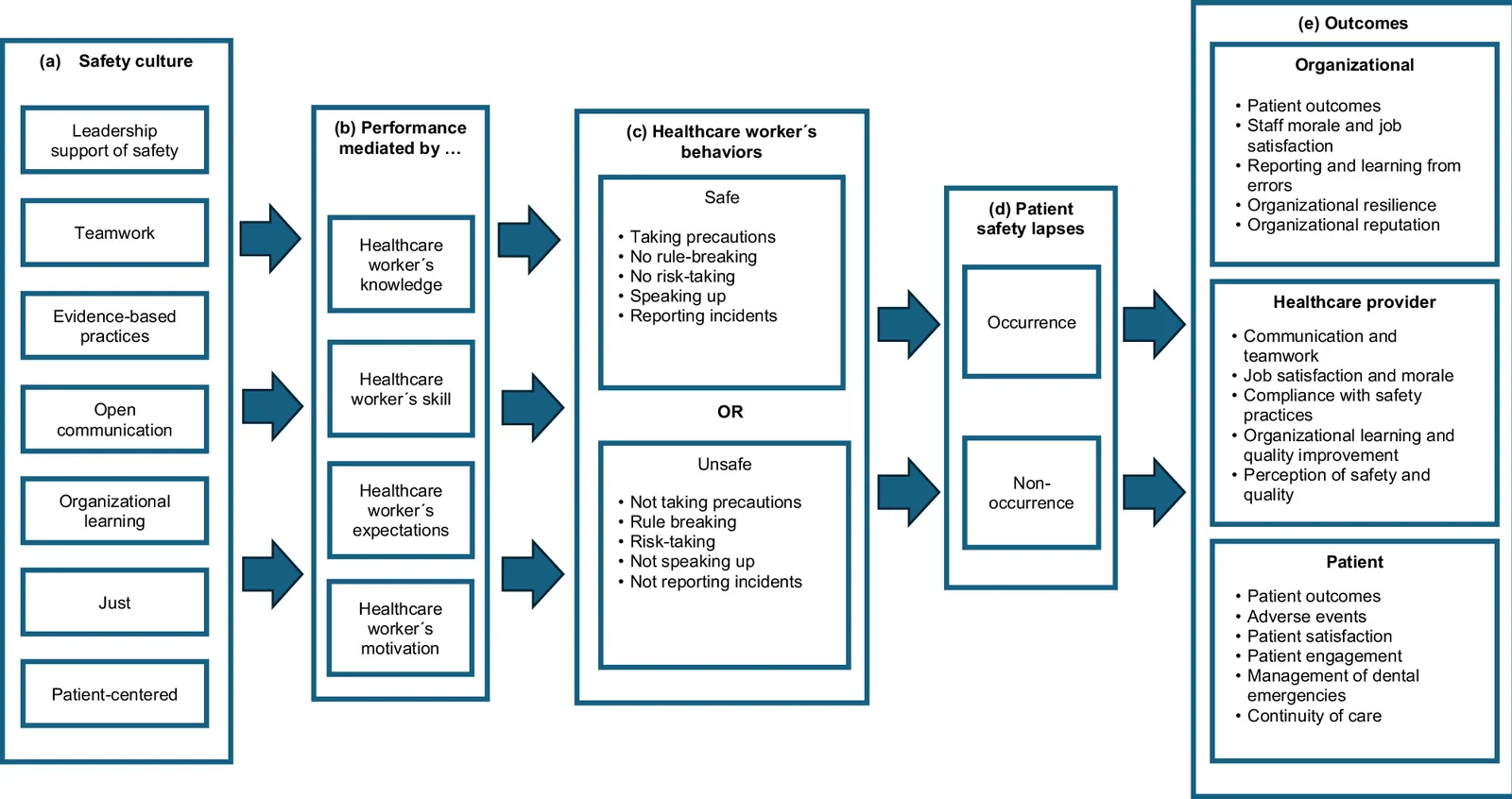
Adaptation of the explanatory model proposed by Flin (2007) for patient safety culture in dentistry. illustrating (**a**) safety culture, (**b**) factors mediating performance, (**c**) healthcare workers’ behaviours, (**d**) patient safety lapses, and (**e**) outcomes.

Since important studies like the Harvard Medical Practice Study [40], the follow-up report “To Err is Human,“ [41] and Resolutions WHA55.18 and WHA72.6.4,9, a variety of approaches have been made to make healthcare safer for patients. Some of these strategies are teaching and training professionals, clinical governance systems, safety standards that are part of the accreditation process, and creating a culture of safety. At the moment, these projects are the most important ones for health systems in countries that are part of the Organization for Economic Co-operation and Development (OECD) [42]. The medical field is starting to understand how important organizational culture is for providing safe and high-quality healthcare [43]. A 2017 meeting with experts selected by official OECD delegates found that patient safety culture is a key factor in improving patient safety [21]. This is because it affects the safety of healthcare facilities and the quality of service as a whole. We modified the explanatory model proposed by Flin R (2007) [44] to illustrate how a culture of patient safety can improve the efficacy of oral healthcare services (Fig. 1).

The term “safety culture” is complex, and various definitions and dimensions have been used in the medical literature [15]. To diminish this variability, Sammer et al. [45] examined the existing empirical literature over an eight-year period and identified seven subcultures or attributes of patient safety culture (Fig. 1a). Table 2 explains why these properties changed in dentistry. The interplay of these attributes, occurring at the organizational or work team level (Fig. 1a), affects the perception and prioritization of safety at the individual level in a clinical ambulatory setting, such as a private dental clinic or a dental school. Figure 1b shows that the overall performance of oral healthcare workers in delivering care is affected by their knowledge and skills, as well as their expectations and motivations about safety. In this way, the behavior of oral healthcare providers will show whether they are doing a good job or a bad job (Fig. 1c). Unsafe practices, such as failing to take precautions or breaking rules, can lead to patient safety lapses, also called patient safety incidents (Fig. 1d). These incidents can have undesirable consequences on both the patient and the oral healthcare provider, as well as on the organization (Fig. 1e).

**Table 2:** Rationale for patient safety subcultures in dentistry.

Subcultures	Rationale for dentistry
1. Leadership	Strong leadership creates the environment for safety through promoting best practices such as infection control, communications and risk management. Leaders advocate for supporting safety committees, reporting systems and training programs that foster the protection of both patient and staff.
2. Teamwork	Safer oral healthcare services depend on the collaboration among dentists, hygienists, assistants, lab technicians and administrators. Everyone in a dental care setting shares the responsibility for patient safety.
3. Evidence-based practices	Consistent and reliable oral healthcare consists of employing evidence-based protocols, clear procedures for emergencies and maintaining strict infection control standards.
4. Communication	Assertive, structured communication is vital for sustaining a safety-focused culture. All clinical and nonclinical staff in oral health clinics and dental schools must communicate clearly and confidently to ensure patients understand and consent to their treatments. Effective protocols reduce misunderstandings, promote consistent care, and improve patient safety.
5. Learning	A culture of learning requires an organizational commitment to continuous improvement and education. Dental practices and schools should learn from everyday mistakes in a non-punitive environment and incorporate performance improvement processes. This supports quality and safety awareness among oral healthcare providers through ongoing education and training opportunities.
6. Justice	A just culture balances individual accountability with recognition of system failures. It ensures adherence to established protocols and care standards, while acknowledging that errors can result from underlying organizational issues in healthcare. In such a culture, trust between staff and leadership is essential. Errors are reported in a non-punitive environment, with leaders trusting staff to prioritize positive patient outcomes.
7. Patient-centeredness	A patient-centered culture in oral healthcare means prioritizing the needs and preferences of patients and their families. It involves empowering them to actively participate in decisions about their treatment journey. Clinicians should provide clear and concise information regarding treatment options, risks, and benefits, enabling patients to make informed choices about their dental care.

In dentistry, creating a culture of patient safety offers many benefits. For example, better communication and coordination between dental staff and between staff and patients can help reduce mistakes that occur when people do not understand or receive conflicting information, which could affect the quality and safety of patient care [31, 46]. Like what happens in medicine, improving communication and coordination between dental care services makes the workplace more unified and efficient, which leads to better patient care [32, 47]. Also, fostering a culture of safety at work is expected to increase dental staff awareness of and knowledge of best practices and patient safety standards, encouraging them to follow these rules [47]. Creating a culture of safety in dental offices is important for making the workplace a supportive place where employees feel comfortable reporting safety problems and near misses [32]. It also encourages team members to talk to each other and give each other feedback, which leads to constant improvement [48]. One more good thing is that it encourages patients to take an active role in their own care. This includes speaking up about their concerns and hopes for the quality, safety, and satisfaction of their care. This builds patients’ trust and confidence in dental care services and their staff, which leads to better loyalty and retention [49, 50].

## Patient safety culture research in dentistry

Unfortunately, there is not enough solid proof about safety climate studies in this field. Our recent data collection on patient safety climate research in dentistry, covering January 1, 2002, to December 31, 2022, identified only nine studies [51]. These studies exhibited significant methodological discrepancies, indicating a more gradual advancement of patient safety climate research in dentistry relative to medicine. Interestingly, even though questionnaires have been used consistently, “safety culture” has become the most popular term. Despite the reported methodological variability, the evidence suggests that patient safety climate levels were predominantly low or neutral. The scores mentioned could have big effects on the patients, the organization, and healthcare providers, as shown in Fig. 1. Nonetheless, there are no studies evaluating the correlations or associations between safety culture scores in dental care environments and the previously mentioned potential outcomes.

The assessment of patient safety climate in dentistry has predominantly used questionnaires adapted from those used in the medical sector. Some of these are the Safety Attitudes Questionnaire (SAQ), the Hospital Survey on Patient Safety Culture (HSOPS), and the Medical Office Survey on Patient Safety Culture (MOSPSC) [51]. But when trying to compare studies and countries, using different measurement tools can be a problem. Consequently, it is imperative to prioritize research in this domain within dentistry. From a research perspective, enhancing patient safety in dentistry requires robust, standardized methods to evaluate the patient safety climate [47]. It is important to get those with expertise to agree on what patient safety culture and climate are and how they work. It is also very important to encourage professionals to share evidence with one another to improve their understanding and make dental practices safer for patients.

## Strategies for enhancing safety culture and safety climate in dentistry

Improving patient safety requires a system in which clinical and non-clinical staff members, patients, and the clinical and non-clinical technologies used in oral care delivery work together to achieve safe oral health care. This, in consequence, requires a strategy that addresses both safety culture and safety climate. The first one reflects the core values, beliefs, and practices that guide both clinical and non-clinical environments, whereas the second addresses how people perceive and apply those values in daily work. In this context, patient safety in dentistry depends not just on technical skills or adherence to rules, but on the complex interplay among individual, team, and organizational factors.

This interaction of elements can be explained through the perspective of Human Factors and Ergonomics (HFE), which views these components as interrelated layers of a sociotechnical system. In this framework, the layers correspond to five main elements or factors involving the individual (e.g., the clinician) who interacts with the tasks, tools, technology, and organizational structures within clinical or nonclinical areas of the dental clinic. Moreover, the performance of each of these main elements both influences and is influenced by the others and may contribute to placing the safety culture and climate at risk [52, 53]. Therefore, understanding these concepts is just as important as understanding the other factors that contribute to patient safety incidents and adverse events. By doing so, solutions can be developed and implemented to strengthen the cultural foundations of safety and enhance the environment experienced by staff and patients during the provision of routine oral health care services [32].

### Organizational commitment and leadership

Achieving patient safety requires strong, continuous leadership and advocacy within the workplace. In doing so, patient safety becomes a priority and is embedded in the organization’s mission and daily work. This helps build a culture of safety in the workplace, making safety central to oral healthcare delivery [54]. Moreover, the implementation of transformational and authentic leadership models contributes to building people´s trust, staying motivated, and keeping making progress [55]. This, in consequence, shapes the culture and the mood that employees feel every day [56] as patient safety, as shown in Fig. 1, is determined by the organization and the individual behaviors of the staff members of the work system. This means that, from an HFE perspective, dental private practices and dental clinics, including those within universities or faculties, are all sociotechnical systems. This means that the way people, tasks, technologies, environments, and organizational frameworks interact is constantly evolving. Therefore, problems in any one area can put patients´ safety at risk [52, 53]. Therefore, once clear organizational goals, rules, and procedures are established, this helps to reduce variations in procedures and processes and demonstrates that safety is the most important priority [57].

Realistic workload planning and management are essential strategies to minimize errors and reduce staff members’ fatigue. Because burnout has been linked to dental errors [37], monitoring workload and supporting staff well-being should also be regarded as part of a patient safety strategy. Yet organizational frameworks that promote open communication and a supportive work environment among staff, both of which reinforce safe practices, are also important [58]. Technology, such as electronic health records and digital event reporting systems, further strengthens an overall strategy to ensure patient safety. However, its potential is fully useful when paired with effective feedback mechanisms to enhance accuracy, consistency, and organizational learning [59, 60]. These strategies should also be accompanied by robust infection control protocols and well-designed clinical environments to integrate patient safety into routine clinical activities [61]. Finally, safety audits, performance metrics, and safety climate assessments can provide dental practices with the means to monitor their progress, identify key areas for improvement, and ensure accountability [21, 31].

### Education and training

Education and training provide staff members and patients with the necessary knowledge and competencies aligned with patient safety practices and the organization’s and leaders’ vision [47]. Through continuing education programs, staff members can improve their skills to prevent errors, strengthen their communication, and teamwork. On the other hand, patient education is just as important as it informs patients about their treatments and encourages them to take an active role in their own care [31]. In this way, training facilitates translating safety culture, an abstract concept, into tangible shared behaviors and practices among the staff members that reflect the concept of safety climate [47].

### Communication and reporting systems

There is a need for effective ways for employees to apply what they learn in training. Strong, non-punitive reporting systems make it easier for people to report mistakes and near misses, turning individual experiences into learning for the whole organization [62]. Feedback systems that close the cycle, so that staff can see what happens after they report something, create an environment of trust and responsibility [63]. Over time, these systems help create a culture where being open and always getting better is normal, not something to be ashamed of.

### Standardization and protocols

Reporting systems often show that practices are not always consistent, but this can be addressed by using standardized procedures and evidence-based recommendations. Developing standard operating procedures and employing checklists demonstrates the organization´s commitment to reliability and rigor and reduces errors. A scoping assessment conducted by Zouaidi et al. [64] found that the use of patient safety checklists in dentistry was associated with fewer adverse events [64]. These tools or devices can help make the workplace stable and safe when utilized on a regular basis. This makes both staff and patients feel secure about the treatment they are receiving [31].

### Teamwork and collaboration

Effective teamwork is also important to patient safety in dentistry. For instance, standardization of procedures and adherence to clinical protocols can only be achieved when all staff members, such as dentists, hygienists, assistants, and office staff, work together toward a shared goal [65]. This should be supported by other strategies, such as simulation training and team-based exercises, to increase staff members’ awareness and readiness [66]. These practices foster a culture of mutual respect and shared responsibility, which contributes to creating an environment in which team members feel supported by their colleagues [67]. Considering that dental care is delivered by teams, the quality of teamwork is likely to influence the quality and safety of the services patients receive. In addition, the size, composition, and stability of dental teams can also affect the dynamics of collaborative work and, consequently, patient safety outcomes [65].

In this scenario, leadership styles are important. Authoritarian or inconsistent leadership tends to inhibit open dialog and collaborative work, whereas supportive and participatory leadership fosters trust and transparent communication [54, 68]. The quality of interpersonal relationships among team members is also important, as high levels of trust, mutual respect, and peer support encourage people to voice concerns and work together to prevent errors [68, 69]. In well-functioning teams, safety is a shared responsibility, and each staff member actively helps create a safe environment. Conversely, poor communication may negatively impact both safety culture and safety climate, contributing to unsafe patient safety practices [32, 54].

### Patient involvement

Patient involvement is another way to integrate safety culture and safety climate. Systematically collecting patient feedback on safety concerns helps align clinical and organizational processes with real-world practice and reveals challenges that staff may overlook [32, 49]. Clear communication with patients about their procedures reduces anxiety, builds trust, and helps professionals respond more effectively to their needs [63, 70]. When a dental office or clinic promotes patient participation, people stop viewing themselves as passive recipients of care and become active partners in their treatment [31, 49].

### Technological solutions

Technology can strengthen the structure of a dental clinic and support the implementation of patient safety strategies, such as integrating reporting systems and using electronic health records (EHRs). In this context, Siriwata et al. [39] proposed the Dental Incident Reporting System (DIRS) to facilitate incident reporting and promote standardization across dental practice [59]. On the other hand, EHRs enhance the accuracy and consistency of healthcare delivery by providing comprehensive, up-to-date patient information, thereby reducing errors and improving coordination among healthcare providers [60, 71]. A recent study from Mensah et al. [72] reported that health professionals are more likely to adopt digital tools for patient safety when they perceive them as reliable and useful [72]. Moreover, EHR-based trigger tools also enable practices to routinely identify patient safety incidents, therefore supporting learning as these events are analyzed [73]. The implementation of these innovations can also foster a work environment in which staff are equipped with the necessary tools to ensure patient safety.

### Continuous improvement and assessment

Patient safety strategies must be routinely improved and assessed to ensure their effectiveness [31, 32]. In this context, regular patient safety audits and the monitoring of performance indicators are useful to determine whether implemented strategies are making progress and to guide any necessary adaptations [47, 57]. The presence of these strategies within a healthcare environment, such as a dental care practice, fosters a culture of accountability and flexibility in which both staff members and patients acknowledge that patient safety is an ongoing journey rather than a one-time achievement [31, 32].

## Conclusion

Patient safety in dentistry is as important as in any other field in healthcare. Available evidence shows that incidents and adverse events do happen, and many can be prevented. Strategies to strengthen patient safety in dentistry are similar to those already developed in medicine and include fostering a culture of safety among all staff involved in providing oral healthcare, whether in private or public clinical practice or in a dental school. However, dentistry also faces the same confusion about the distinction between safety culture and safety climate. On one hand, safety culture encompasses the shared values and beliefs that guide behavior, while safety climate refers to how staff and patients perceive everyday practices. These perceptions, however, do not emerge in isolation; they are the product of the complex interactions within the system where dental care takes place. From HFE perspective, dentistry is a sociotechnical system shaped by dynamic interactions among people, tasks, technologies, environments, and organizational frameworks. Consequently, any deficiency in training, teamwork, leadership, staff well-being, or infrastructure can weaken this system at any point and compromise overall safety.

This literature review identifies key strategies to strengthen safety culture: visible leadership, continuous education, learning from incident reporting systems, standardized procedures, effective teamwork, patient involvement, technology support, and continuous evaluation. However, these strategies are comprehensive; no single intervention, on its own, is likely to be sufficient. Instead, they must be incorporated through a coordinated, context-sensitive approach tailored to the diverse structures and needs of dental care systems. Therefore, future initiatives should focus on employing robust standardized methods for assessing safety culture and safety climate, adapted to different oral healthcare settings. These should also be accompanied by stronger collaboration among researchers, policymakers, and practitioners, particularly from low- and middle-income economies. At its core, patient safety is a shared professional value and not merely a protocol to follow. When it is embedded in daily practice through organizational commitment, teamwork, and patient engagement, it can reduce adverse events and enhance the quality of care for both patients and staff members.

## Data Availability

Not applicable. This article does not include any primary data.
